# Hydrophobic interaction chromatography resolves extracellular vesicle fractions with distinct lipidomic signatures

**DOI:** 10.1038/s41598-026-50782-x

**Published:** 2026-05-20

**Authors:** Michał Młynarczyk, Wiktoria Więckowska, Mariusz Belka, Raphael Ewonde Ewonde, Jagoda Mantej, Mikołaj Klimczuk, Felicja Gajdowska, Jorge Matinha-Cardoso, Paula Tamagnini, Danuta Gutowska-Owsiak, Paulo Oliveira, Sebastiaan Eeltink, Weronika Hewelt-Belka

**Affiliations:** 1 Department of Analytical Chemistry, Faculty of Chemistry, Gdańsk University of Technology, Gdańsk, Poland; 2 Department of Pharmaceutical Chemistry, Medical University of Gdańsk, Gdańsk, Poland; 3 Department of Chemical Engineering, Vrije Universiteit Brussel, Brussels, Belgium; 4 Laboratory of Experimental and Translational Allergology and Pneumology, Medical University of Gdańsk, Gdańsk, Poland; 5 Laboratory of Experimental and Translational Immunology, Intercollegiate Faculty of Biotechnology of University of Gdańsk and Medical University of Gdańsk, University of Gdańsk, Gdańsk, Poland; 6 MCbiology Doctoral Program, ICBAS – School of Medicine and Biomedical Sciences Abel Salazar, University of Porto, Porto, Portugal; 7 CIIMAR – Interdisciplinary Centre of Marine and Environmental Research, University of Porto, Matosinhos, Portugal; 8 i3S - Instituto de Investigação E Inovação Em Saúde, University of Porto, Porto, Portugal; 9 Department of Biology, Faculty of Sciences, University of Porto, Porto, Portugal; 10 Present Address: RezonBio, Gdańsk, Poland; 11 Present Address: Department of Chemistry, Biosystems Research Complex, Clemson University, Clemson, SC USA; 12 Present Address: Institute for Research in Biomedicine (IRB Barcelona), The Barcelona Institute of Science and Technology, Barcelona, Spain

**Keywords:** Hydrophobic interaction chromatography (HIC), Extracellular vesicle, Lipidomics, Surface hydrophobicity, Human milk extracellular vesicles, Biochemistry, Biological techniques, Biophysics, Biotechnology, Chemistry

## Abstract

Current extracellular vesicle (EV) isolation workflows are dominated by size- and density-based approaches, which provide limited insight into surface chemical properties of vesicular particles. Here, we report a hydrophobic interaction chromatography (HIC) workflow for resolving EV fractions along differences in membrane interfacial hydrophobicity. Using a commercially available HIC column, reproducible fractionation of EV samples was achieved, yielding discrete fractions that differed in retention behaviour and lipid composition. Transmission electron microscopy (TEM) showed vesicle-like nanoparticles with EV-consistent morphology, indicating preservation of vesicle integrity during HIC. Lipidomic profiling by reversed-phase liquid chromatography coupled with quadrupole time-of-flight mass spectrometry (RP-LC-Q-TOF-MS) revealed fraction-specific differences in lipid composition. Importantly, we demonstrate the feasibility of direct injection of pre-cleaned biofluids onto the HIC column, enabling fractionation of nanoparticle populations containing EVs without prior ultracentrifugation. Furthermore, an operational interfacial hydrophobicity index derived from lipidomic data showed a clear correlation with HIC retention, providing an orthogonal compositional descriptor consistent with the proposed fractionation mechanism. Together, this hydrophobicity-based strategy introduces a previously unexplored physicochemical dimension to EV analysis, revealing chemically structured heterogeneity that is not accessible using conventional separation strategies. The workflow provides a practical framework for fractionating EVs in a manner directly relevant to lipidomic profiling and studies of EV membrane chemistry.

**Supplementary Information:**

The online version contains supplementary material available at 10.1038/s41598-026-50782-x.

## Introduction

Extracellular vesicles (EVs) are lipid bilayer-enclosed nanoparticles actively released by cells and play critical roles in intercellular communication as important transporters of bioactive molecules, including proteins, lipids, DNA, RNA, and various metabolites^1^. EVs influence immune modulation, tissue repair, and disease progression, particularly in cancer and neurodegenerative disorders^2^. EVs are found in many biological fluids, including blood, milk, urine, and cerebrospinal fluid, making them easily accessible and widely explored as a non-invasive source of molecular information for disease diagnostics and monitoring, including their use in a liquid biopsy^3^. Despite recent advances, the isolation of EVs remains technically challenging. Their nanometric size and compositional heterogeneity, together with the frequent co-isolation of lipoproteins, protein aggregates, and other nanoscale particles, compromise purity and quantitative interpretation^4–8^. Moreover, most isolation methods do not resolve EV subpopulations by membrane surface chemistry. Without this selectivity, differences in lipid composition are averaged across particles, biasing lipidomic measurements, potentially masking diagnostically relevant signals, and limiting mechanistic interpretation of EV function. Because outer-leaflet lipid classes shape the aqueous-facing interface, they provide a chemically meaningful axis for EV fractionation.

The choice of isolation technique, each exploiting distinct physicochemical features of EVs, such as size, density, or surface markers, has a strong impact on both the purity and recovery of EVs. Ultracentrifugation (UC) is widely used for EV fractionation, as it enables reproducible differentiation based on size and density without the use of labeling or affinity reagents^8^. However, it holds several limitations, since it is time-consuming, requires expensive equipment, and may lead to co-isolation of protein aggregates or other non-vesicular contaminants, potentially compromising the purity and integrity of the isolated EVs. Size-exclusion chromatography (SEC)^9–11^ and asymmetric flow field-flow fractionation (AF4) also operate on the principle of hydrodynamic volume^12,13^; however, their resolving power is limited. Immunoaffinity capture enables selective isolation of EV-associated subpopulations by targeting specific surface markers using antibodies, offering high specificity and purity^14,15^. Nonetheless, this method is constrained by low throughput and high cost.

Hydrophobic interaction chromatography (HIC) has emerged as an alternative approach for isolating EVs from biological fluids such as human plasma^16^ and urine^17–19^. In contrast to size- and density-based techniques, HIC exploits differences in exposed surface chemistry under high ionic strength conditions. The Marcus group demonstrated the feasibility of isolating small EVs from complex biofluids using a unique HIC stationary phase composed of capillary-channeled polymer fibers based on polyethylene terephthalate^16,19,20^ and nylon-6^18^. HIC exploits the hydrophobic properties of the EV membrane surface, which are determined by the lipid bilayer and other components embedded within the membrane.

The aim of this study was to examine whether HIC can be used to resolve EV fractions based on differences in lipid membrane surface hydrophobicity, a property not exploited by existing isolation techniques. Access to this chemical dimension is particularly relevant for lipidomics-driven EV studies, where averaging effects can obscure chemically and biologically meaningful variation. HIC was applied to fractionate EV samples, and the resulting fractions were analysed by reversed-phase liquid chromatography coupled with quadrupole time-of-flight mass spectrometry (RP-LC-Q-TOF-MS) to determine their lipid composition. EV samples obtained by UC were used as reference material for HIC-based fractionation, enabling controlled assessment of EV lipid heterogeneity. The feasibility of using a commercially available HIC column for EV fractionation, with particular focus on preserving vesicle integrity, was assessed. The possibility of direct injection of pre-cleaned biological samples into the HIC system was also demonstrated. Importantly, the workflow relies on standard HPLC instrumentation and a commercially available HIC column, supporting straightforward adoption in routine analytical laboratories.

## Results and discussion

### Extracellular vesicle fractionation by HIC: insights into lipidomic heterogeneity across resolved EV fractions

**Fig. 1:**
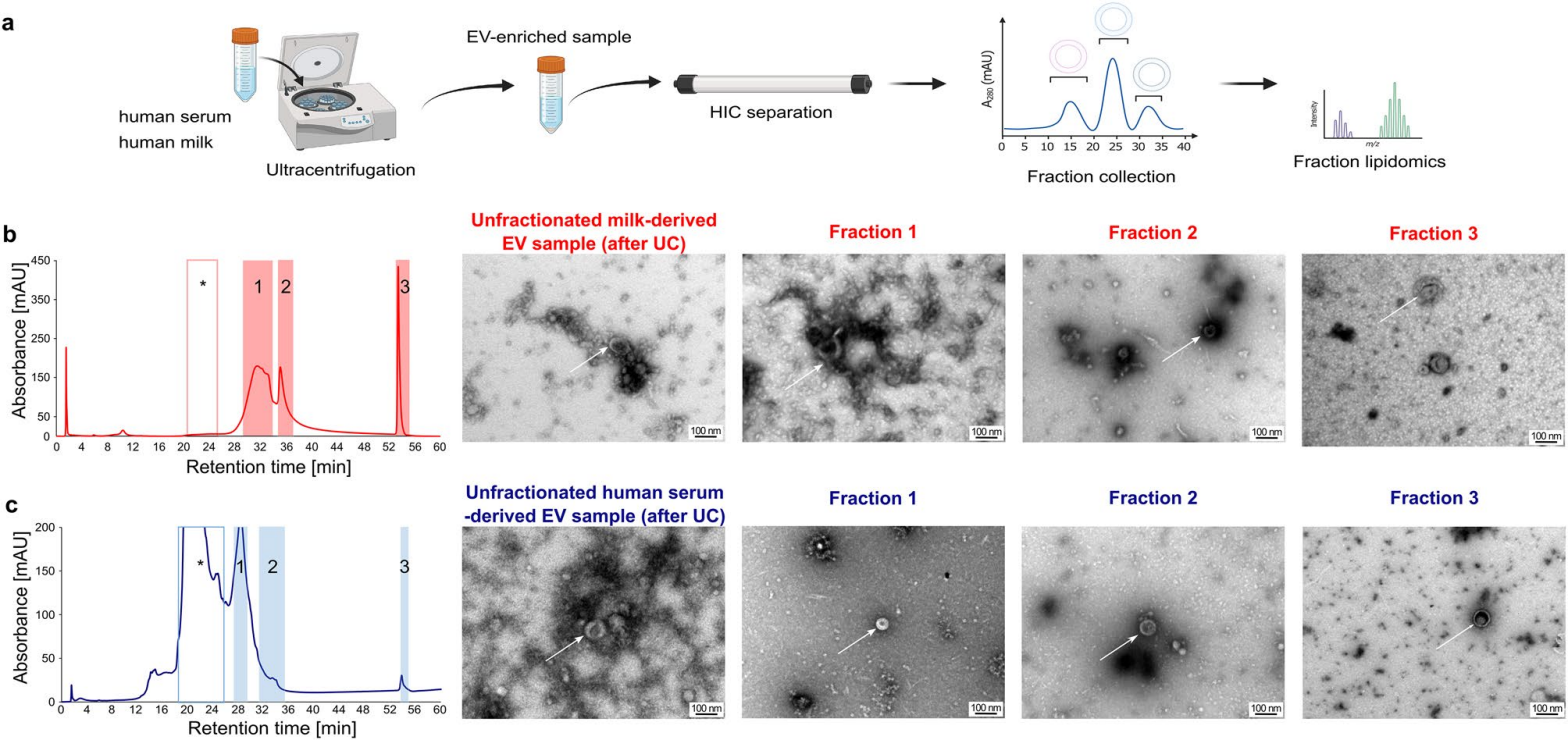
Hydrophobic interaction chromatography (HIC)–based fractionation of extracellular vesicle (EV) samples from human milk and serum. (**a**) Schematic overview of the experimental workflow. EV samples obtained from human serum or human milk by ultracentrifugation (UC), were subjected to HIC separation, followed by fraction collection and lipidomic analysis. (**b**) Representative HIC chromatogram of a milk-derived EV sample after UC, with indicated fraction collection windows, and corresponding negative-stain transmission electron microscopy (TEM) images of the unfractionated sample and selected HIC fractions. (**c**) Representative HIC chromatogram of a serum-derived EV sample after UC, with corresponding TEM images of the unfractionated sample and selected HIC fractions. Scale bars, 100 nm. Fractions marked with an asterisk (*) did not contain EV-like particles detectable by negative-stain TEM. Representative chromatograms and TEM images are shown. Arrows indicate EVs.

The potential of HIC to resolve EVs based on differences in surface properties (as both lipids and proteins embedded in the lipid membrane) was assessed. Since there are no commercially-available HIC columns with mesopores large enough to accommodate EVs (100–200 nm range), non-porous stationary phase was selected. The 5 µm particle size offers sufficient interstitial flow paths for particles in the column, enabling EV elution while maintaining vesicle integrity. Figure 1a displays an overview of the experimental workflow used for the HIC fractionation of EVs. EV samples isolated from human milk and human serum by UC were used as reference samples. The initial salt concentration at the gradient start was set to 1.5 M to ensure adequate retention and prevent sample breakthrough, while avoiding visible precipitation under the applied conditions. Lower ammonium sulfate concentrations resulted in insufficient retention of nanoparticles, whereas higher concentrations increased the risk of excessive retention of matrix constituents and protein precipitation. UV detection at 210 nm was used for chromatographic monitoring and fraction triggering. All separations were performed on a conventional analytical HPLC system equipped with UV detection and automated fraction collection. The robustness of the method was verified by repeated injections (n = 5) of the human milk EV sample, which showed good retention-time repeatability and overall stable UV signal across replicate injections (Supplementary Information, Fig. S1 and Table S1). The observed peak broadening is expected for nanoscale particles and likely reflects slow mass-transfer kinetics and increased dispersion relative to small molecules. No significant carry-over was observed between consecutive runs, as verified by blank injections.

Collected fractions and the corresponding unfractionated reference samples were analyzed by transmission electron microscopy (TEM), nanoparticle tracking analysis (NTA), and Western blotting (Supplementary Information, Figs. S2–S3) to assess vesicle-like morphology, particle size distributions, and the presence of co-eluting lipoproteins. Background analysis of phosphate-buffered saline (PBS) solution was performed as a control to assess instrumental noise, confirming that the detected signals originated from nanoparticle-containing samples.

Representative HIC separations of EV samples are shown in Fig. 1b-c. The chromatogram of the human milk EV sample shown in Fig. 1b marks three time intervals designated for fraction collection (fractions 1–3). Each of these fractions was analyzed by TEM, revealing nanoparticles with a characteristic “cup-shaped” morphology, consistent with EVs. A distinct late-eluting peak (t_r_ = 53 min), containing EV-like particles (fraction 3), was observed, consistent with earlier HIC studies reporting strong EV-stationary-phase interactions^17^. No EV-like vesicles were detected by negative-stain TEM in the fraction marked with an asterisk. NTA revealed size differences among the collected fractions: mean particle diameters were 125 nm in fraction 1; 137 nm and 196 nm in fraction 2 (two size populations); and 161 nm in fraction 3 (see Fig. S2a).

Figure 1c shows the representative HIC chromatogram of EVs from human serum, with highlighted regions indicating the fractions collected for TEM imaging. EV-like vesicular structures were detected by TEM in fractions 1 to 3. A strong early UV signal (12–24 min) reflects abundant proteins and lipoproteins, as confirmed by Western blot analysis and protein assays (Supporting Information, Fig. S3b and c). The final EV peak exhibits lower intensity in comparison to human milk EVs, because only 1.2 × 10^8^ particles were loaded for the EV serum sample, versus 1.1 × 10^11^ for the EV milk sample.

To account for differences in particle concentration across fractions, Western blot signals were additionally normalized to particle number (expressed per 1 × 10^7^ particles; Fig. S4 a-b), enabling comparison of marker signals independently of total sample loading. Following normalization, differences in marker distribution across fractions remained evident. Distinct profiles of EV-associated (CD63, flotillin) and non-EV-associated (ApoA1) markers were observed across fractions, indicating compositional heterogeneity of nanoparticle populations resolved by HIC. While the CD63 was present in every fraction, flotillin was less pronounced, particularly in fractions derived from serum, which suggests the presence of different EV subpopulations in biofluids^21^. ApoA1 signal, consistent with high-density lipoprotein particles, remained prominent across fractions (Figs. S2c and S3c), indicating a substantial contribution of lipoprotein-associated material, as expected for lipoprotein-rich biofluids in the absence of a dedicated lipoprotein removal step prior to HIC. Although such signals are typically considered contamination in EV preparations derived from biofluids using differential UC^22^, emerging evidence indicates that ApoA1 can also associate with the EV corona^23^. Thus, the observed signal may originate from either co-isolated lipoproteins or ApoA1 bound to the EV surface. These observations emphasize that HIC resolves EV-containing nanoparticle populations along an interfacial chemistry axis, and co-elution can occur for non-EV nanostructures with similar surface properties.

### Injection of pre-cleaned biofluids

**Fig. 2:**
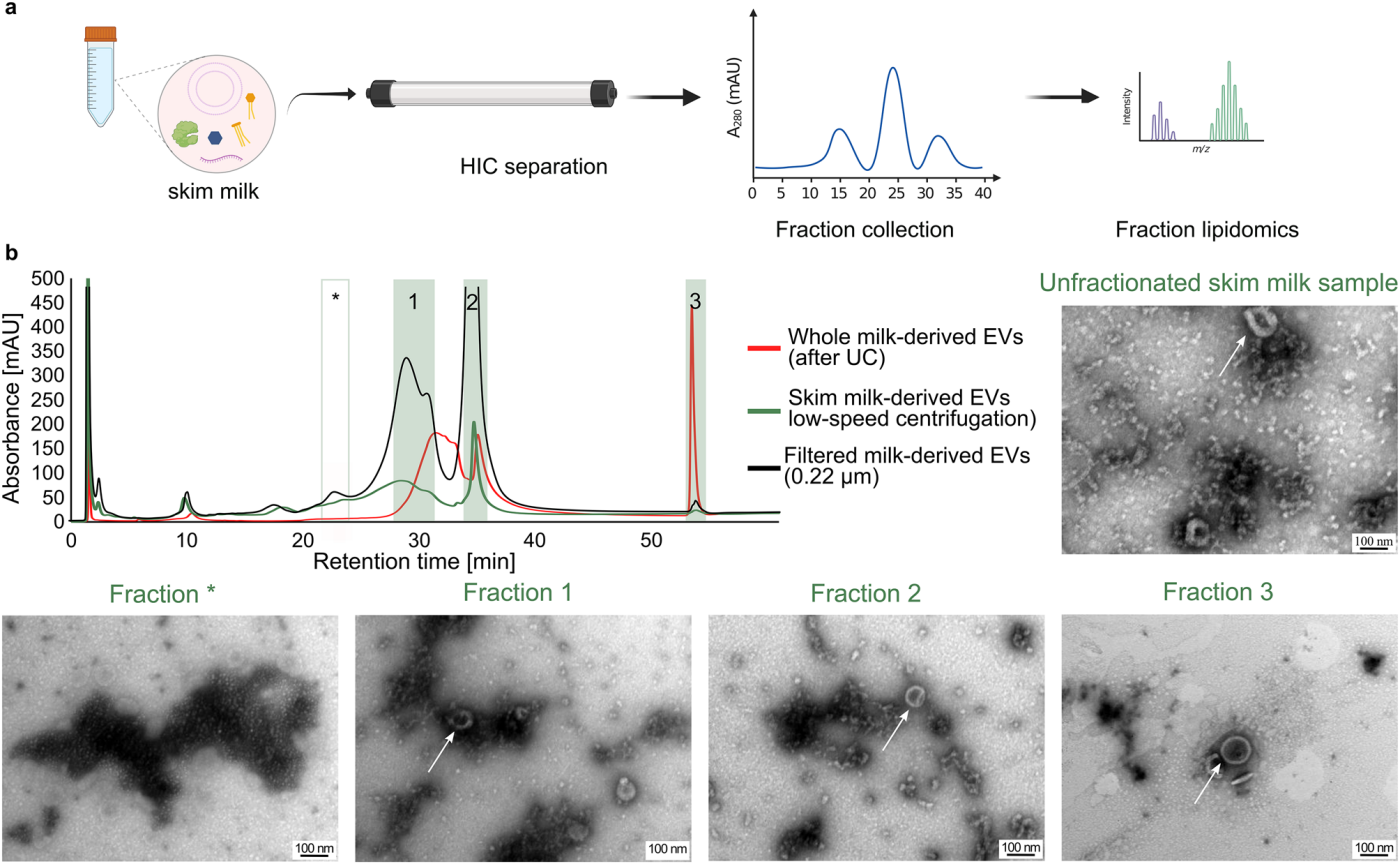
Direct injection (no UC) hydrophobic interaction chromatography (HIC) enables resolving extracellular vesicle (EV)–containing fractions from human milk without prior UC. (**a**) Schematic overview of the direct-injection HIC workflow. Human milk samples are pre-cleaned by low-speed centrifugation or filtration and injected directly into a HIC column, followed by automated fraction collection and downstream characterization of EV-containing fractions. (**b**) Overlaid HIC chromatograms obtained for different human milk preparations: EVs isolated by UC (red), skim milk obtained after low-speed centrifugation at 2310 × g for 10 min (green), and milk filtered through a 0.22 µm membrane (black). Shaded regions indicate fractions collected for further analysis. (**c**) Representative transmission electron microscopy (TEM) images of unfractionated samples (before HIC separation) and corresponding HIC fractions. EV-like vesicles are observed in fractions 1–3 for both UC-processed and directly injected skim milk samples. The fraction marked with an asterisk (*) contained aggregated nanoparticle structures and was not analysed further in this study. Scale bars: 100 nm. Arrows indicate EVs.

An overview of the experimental workflow for direct injection of pre-cleaned biofluids and subsequent HIC fractionation is shown in Fig. 2a. Direct injection experiments were primarily explored for human milk, which can be collected non-invasively in larger volumes, enabling extensive method development, and exhibits higher particle abundance compared to serum. Figure 2B shows overlaid chromatograms of EV separations from three preparations of human milk samples: (1) whole milk subjected to UC, (2) skim milk obtained after low-speed centrifugation at 2310 × *g* for 10 min, and (3) milk filtered through a 0.22 µm membrane. Pre-cleaning steps (centrifugation or filtration) were applied to remove large particles and debris (fat globules (~ 4–5 µm) and cells) that could adversely affect the column performance. Whole-milk EVs were purified by UC prior to HIC, whereas skim- and filtered-milk samples were directly loaded onto the column without prior EV isolation. UC-enriched EV sample showed a prominent late-eluting peak at ~ 55 min.

This study demonstrates that direct injection of human skim milk enables the collection of fractions containing EV particles, as confirmed by TEM images of fractions 1–3 (Fig. 2B). Additional characterization of skim milk-derived fractions and unfractionated skim milk by NTA and Western blot analysis is provided in the Supplementary Information (Fig. S5). The fraction marked with an asterisk contained globular structures with EV-like morphology that were not observed in the UC-processed sample. Their origin and identity remain unclear, and they were not studied further. As in UC-enriched samples, ApoA1 signal remained prominent, confirming the contribution of lipoprotein-associated material under direct-injection conditions (Fig. S4c). Comparison of chromatographic profiles (20–35 min elution window) for milk samples subjected to UC or low-speed centrifugation indicates that the observed composition depends on the applied sample processing method. Moreover, filtration through a 0.22 µm membrane markedly reduced the peaks eluting at ~ 30 and ~ 35 min relative to skim milk, suggesting that these peaks include particles larger than the filter cut-off or by aggregates retained by the membrane. Direct injection used substantially smaller input volumes (10 µL skim milk per injection) than UC-enriched preparation (derived from 30 mL milk), which likely contributes to the lower intensity of late-eluting EV-containing peaks. Nevertheless, EV-containing fractions could be reproducibly detected and collected, supporting the feasibility of direct-injection HIC as a practical screening and fractionation approach.

Under the current analytical-scale conditions, the amount of material recovered per individual fraction is limited, and multiple injections were therefore pooled for downstream analysis. Accordingly, HIC is applied here as an analytical separation and fractionation approach rather than as an EV enrichment/concentration method. Increasing sample load and exploring alternative column formats may improve throughput in future applications.

### Lipid profiling of HIC-separated EV fractions

**Fig. 3:**
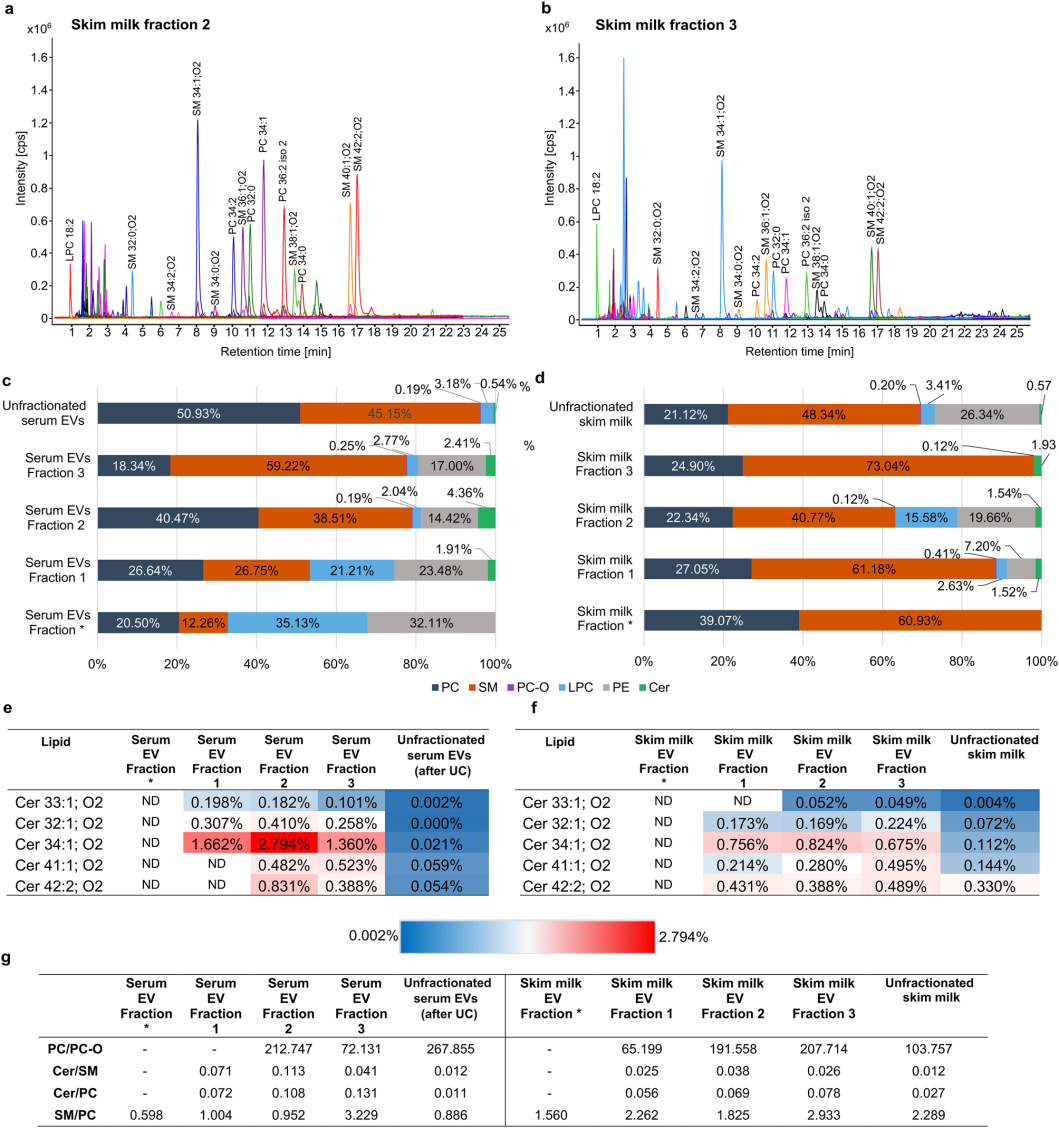
Comparative lipidomic profiling of EV-containing fractions separated by hydrophobic interaction chromatography (HIC). Extracellular vesicle (EV)–containing fractions obtained by HIC from human serum and human skim milk were analysed by LC–MS-based lipidomics. Serum EVs were purified by ultracentrifugation (UC) prior to HIC separation, whereas skim milk was directly injected onto the HIC column without prior EV isolation. The elution ranges corresponding to the analysed EV-containing fractions are indicated on the HIC chromatograms shown in Figs. 1B and 2B, respectively. Panels (**a**) and (**b**) show representative extracted-ion chromatograms of selected lipid species detected in collected fractions from human skim milk. Panels (**c**) and (**d**) present the relative abundance (% of total lipids) of major lipid classes in each HIC fraction and in the corresponding unfractionated samples for serum- and milk-derived EVs, respectively. Panels (**e**) and (**f**) show heat maps of individual ceramide species abundances (expressed as % of total lipids), with colour gradients indicating relative abundance from lowest (blue) to highest (red). Panel (**g**) shows ratios between selected lipid classes across HIC fractions and unfractionated samples.

To assess whether HIC-separated fractions differ at the level of membrane composition, lipidomic profiling was performed on collected fractions. Major structural lipids of EV membranes were profiled in HIC-separated EV-containing fractions obtained from human serum and human milk using LC–MS-based lipidomics (Supporting Information, Table S2). The dominant classes included diacylglycerophosphocholines (PC) and their ether analogues (PC-O), lysoglycerophosphocholines (LPC), ceramides (Cer), and sphingomyelins (SM). Representative chromatograms of HIC separated fractions 2 and 3 from skim milk are shown in Fig. 3b. Lipid abundances are presented as relative proportions (% of total lipids), enabling comparison of compositional differences across fractions despite differences in collected fraction volumes. This approach normalizes lipid profiles to the total lipid pool and avoids potential bias associated with normalization to particle number or protein content, as particle counts may be affected by the presence of non-vesicular components or aggregates. Estimated particle numbers associated with each lipidomics sample are provided in the Supplementary Information (Table S3).

Lipidomic profiling revealed pronounced differences in lipid class abundance and composition across the collected fractions, as well as relative to the unfractionated (not separated by HIC) samples (Fig. 3).

Because SM and PC are commonly enriched in the outer leaflet of mammalian membranes, even moderate shifts in their relative abundance can substantially alter effective membrane surface hydrophobicity^24–26^. Variations in class ratios (Fig. 3c-d) indicate shifts in membrane organization among subpopulations that could help explain their differential HIC retention. In particular, changes in the sphingomyelin-to-phosphatidylcholine (SM/PC) ratio across fractions further illustrate fraction-specific differences in membrane composition, especially for serum-derived EV samples. Such variations in lipid composition are known to influence membrane organization, for example, exosomal membranes enriched in sphingomyelin and ether lipids tend to form more ordered and rigid structures^25^. In milk-derived EV-containing fractions, differences were less pronounced, likely due to co-eluting non-EV components such as lipoproteins, as confirmed by Western blot analysis. Importantly, the lipidomic variation captured by HIC separation can correspond to biologically relevant differences in membrane architecture, such as fluidity, rigidity, and curvature, which in turn may be linked to different membrane formation pathways, including ceramide-driven inward budding or plasma membrane shedding, processes known to generate vesicles with different molecular cargos and biological activities^27–29^. Notably, a repertoire of lipid species was also detected in fractions where EV-like particles were not visualized by TEM, indicating the presence of lipid-containing nanoparticles below imaging detection limits or originating from non-vesicular lipid nanoparticles.

Analysis of individual lipid species further revealed distinct enrichment patterns across HIC resolved fractions (Supplementary Information, Fig. S6). Several lipid species showed consistent enrichment relative to unfractionated samples, most prominently ceramides (Fig. 3e-f). Given that ceramides are a characteristic and abundant component of EV membranes, their enrichment is consistent with the presence of EVs in these fractions^29–31^. Beyond biogenesis, elevated ceramide levels have been associated with EV-mediated functional effects, such as neuronal apoptosis via PAR-4 cargo^32^ and endothelial inflammation^33^. Other membrane lipids also influence EV biology, as shifts in sphingomyelin, and ether-linked phospholipids have been linked to disease-relevant functions such as neuroinflammation, endothelial activation, metabolic stress signaling, and tumor-associated immune modulation^33–37^. Together, these results demonstrate that HIC fractionation preserves and reveals chemically meaningful differences in EV membrane composition that are otherwise masked in unfractionated EV samples. Notably, the achieved separation enables resolution of multiple EV-containing fractions, extending beyond the isolation of a single EV fraction typically reported in previous HIC studies. Further improvements in chromatographic design may enable even higher resolution of EV-associated heterogeneity, including exploration of alternative stationary phase chemistries and multidimensional separation approaches (e.g., column coupling), which may further enhance separation of EV-associated heterogeneity.

### Linking EV membrane lipid composition to HIC retention via an operational interfacial hydrophobicity index (H_EV_)

To rationalize differences in HIC retention among EV-containing fractions, an operational interfacial hydrophobicity index (*H*_EV_) was defined for the outer membrane leaflet, *i.e*., the interface expected to contact the butyl stationary phase under ammonium sulfate HIC conditions. Given the lack of an established vesicle hydrophobicity scale, we propose a weighted mean over lipid species with class-level weights, according to Eq. (1):

1 $${H}_{\mathrm{E}\mathrm{V}}=\frac{{\sum}_{\mathrm{i}=1}^{n}\left(HG{I}_{\mathrm{c}\mathrm{l}\mathrm{a}\mathrm{s}\mathrm{s}}\times {f}_{\mathrm{c}\mathrm{l}\mathrm{a}\mathrm{s}\mathrm{s}}\times {C}_{\mathrm{m}\mathrm{o}\mathrm{l},\mathrm{i}}\right)}{{\sum}_{\mathrm{i}=1}^{n}\left({f}_{\mathrm{c}\mathrm{l}\mathrm{a}\mathrm{s}\mathrm{s}}\times {C}_{\mathrm{m}\mathrm{o}\mathrm{l},\mathrm{i}}\right)}$$

Equation 1 represents a weighted mean of class-level hydrophobicity values, in which each lipid’s contribution to the overall interfacial hydrophobicity is proportional to both its molar concentration and the estimated extent of its exposure to the aqueous phase. The denominator normalizes for total outer-leaflet lipid content, yielding a dimensionless, composition-dependent descriptor that can be compared across fractions. For each lipid species i, the calculation combines three quantities: (i) the class-specific headgroup hydrophobicity index (*HGI*_class_) describing the inherent polarity of the lipid headgroup; (ii) the outer-leaflet exposure fraction (*f*_class_), representing how much of that lipid class is exposed on the vesicle surface; and (iii) the molar concentration (*C*_mol,i_), reflecting the quantitative abundance of that species. *HGI*_class_ and *f*_class_ are class-level parameters (0–1) applied to all species within a given lipid class and were defined a priori based on literature describing headgroup hydration and membrane asymmetry. The headgroup-based *HGI*_class_ weights are scaled to follow known trends in interfacial polarity and dielectric response and were assigned: PC = 0; PE = 0.3; SM = 0.5; Cer = 1.0^38,39^. Ether analogues of PC and PE, as well as lysoglycerophospholipids (LPC and LPE), were assigned the *HGI*_class_ of their respective headgroup class. Outer-leaflet exposure fractions (*f*_class_) were defined based on reported membrane asymmetry in mammalian membranes: PC, PC-O, and LPC = 1.0; SM = 1.0; PE and PE-O = 0.2; Cer = 0.5^40,41^. In this framework, *HGI*_class_ values approaching 1 correspond to lipid classes with higher interfacial hydrophobicity (lower headgroup polarity), whereas values near 0 indicate more hydrophilic headgroups. Similarly, *f*_class_ values approaching 1 represent lipid classes predominantly exposed at the outer membrane leaflet, while lower values indicate partial or limited surface exposure. This formulation ensures that only the fraction of each lipid class accessible to the chromatographic surface contributes to *H*_EV_, while more abundant or more hydrophobic headgroups exert proportionally greater influence. Including chain-level descriptors (total carbon number, double bonds, and unsaturation ratios in fatty acyl substituents) did not further improve the model once headgroup class and leaflet exposure were included, consistent with HIC sensitivity to exposed interfacial chemistry under high ionic strength conditions. In practice, the EV surface may also be partially covered with proteins and glycans, a factor not accounted for in the current model. Despite this simplification, *H*_EV_ captured the chromatographic retention in HIC and correlated strongly with the logarithmic retention factor *(log k*_HIC_*)* (R^2^ = 0.9917; Fig. S7). Although evaluated on a limited number of fractions, the monotonic relationship is consistent with interfacial lipid composition contributing to HIC retention under these conditions. *H*_EV_ increased monotonically across the EV-containing fractions, in line with the observed elution order. This relationship indicates that the proposed index is consistent with interfacial lipid composition being a major contributor to retention under hydrophobic interaction conditions. Because *H*_EV_ was computed from independently measured lipid compositions using a priori class and leaflet-exposure weights (not fitted to the retention data), the observed correlation supports a lipid-driven contribution to retention under these HIC conditions. Fractions enriched in sphingomyelin and ceramides (higher *HGI*_class_ and partial outer-leaflet exposure) showed higher *H*_*E*V_ and longer retention, whereas fractions enriched in PC and LPC exhibited lower *H*_*E*V_ and eluted earlier. Overall, the agreement between *H*_EV_ and log *k*_HIC_ supports a lipid-driven contribution to retention under high-salt butyl HIC conditions. The *H*_EV_ framework therefore provides a mechanistically interpretable and computationally tractable link between lipid composition and chromatographic retention. The resulting index provides a straightforward, reproducible measure of vesicle surface hydrophobicity that can be calculated directly from LC–MS lipidomics data and compared with experimental retention factors in HIC.

## Conclusions

Hydrophobic interaction chromatography introduces surface hydrophobicity as an orthogonal physicochemical dimension for the fractionation of EV samples, complementing existing size- and density-based approaches. The applied workflow enables resolution of multiple EV-containing fractions beyond previous HIC studies. By chemically selective separation, HIC allows fraction-resolved lipidomic profiling and reveals membrane lipid heterogeneity that remains inaccessible to conventional workflows. Such molecular resolution is essential for elucidating EV lipidome heterogeneity, as membrane lipid composition directly influences vesicle stability, cargo encapsulation, cellular uptake, and subsequent biological properties. The correlation between lipidomics-derived interfacial hydrophobicity and HIC retention is consistent with a substantial lipid-dependent contribution to retention under ammonium sulfate/butyl HIC conditions. Importantly, the feasibility of direct injection of pre-cleaned biofluids demonstrates that HIC can be implemented as a practical, sample-sparing analytical tool for EV studies. Together, this work establishes HIC as a robust framework for investigating EV-associated membrane chemistry and chemical heterogeneity in complex biological samples. Because the workflow relies on commercially available HIC columns and standard HPLC instrumentation with automated fraction collection, it is readily transferable to routine analytical laboratories. By preserving lipid-level heterogeneity during fractionation, this approach provides an analytical basis for future studies exploring links between EV membrane composition and biological function. In addition to lipid composition, other surface-exposed components such as proteins may also contribute to interfacial properties and could be explored in future studies using complementary omics approaches.

## Methods

### Human samples and ethics approval

All experiments involving human samples were performed in accordance with relevant guidelines and regulations, including the principles of the Declaration of Helsinki. EV samples were characterized in line with MISEV guidelines^42^ using Nanoparticle Tracking Analysis (NTA), Western blotting (WB), and negative-staining transmission electron microscopy (TEM). Human whole blood for serum isolation was provided by the Regional Blood Centre in Gdańsk under bioethical committee approval no. NKBBN/621-547/2020. Human milk samples were obtained within a study approved by the Ethics Committee of the Medical University of Gdańsk (NKBBN/389/2019), as previously described^43^. The present work used aliquots of these previously collected samples, with ethical permission covering secondary analyses. Written informed consent was obtained from all donors prior to sample collection (both blood and milk), and all samples were anonymized prior to analysis.

### Isolation of small EVs from human serum

Small extracellular vesicles (sEVs) were isolated from ~ 30 mL human serum by differential UC. The initial clarification step involved centrifugation at 3000 × *g* for 30 min to remove cells and larger debris. The supernatant was then transferred to clean tubes and subjected to a second centrifugation at 10,000 × *g* for 30 min to further eliminate remaining cellular debris and larger vesicles. UC was performed using a fixed-angle rotor (Fiberlite F37L-8 × 100) and compatible polycarbonate bottles. Following the 10,000 × *g* spin, the supernatant was filtered through a 0.22 µm syringe filter and subjected to UC at 100,000 × *g* for 2 h to pellet the small extracellular vesicles. The resulting pellet was washed by resuspension in ice-cold 0.1 µm-filtered phosphate-buffered saline (PBS) and centrifuged again at 100,000 × *g* for 2 h under identical conditions. The final sEV pellet was suspended in 1 mL of 0.1 µm-filtered PBS and stored at − 80 °C until further analysis.

### Isolation of small EVs from human milk

Approximately 30 mL of human milk (HM) were used for the isolation of small EVs using a UC protocol. All steps were carried out at 4 °C. First, the HM sample was centrifuged at 3000 × *g* for 15 min to obtain the skim milk fraction. The fraction was then filtered through a 0.45 µm membrane. This was followed by a second centrifugation at 3000 × *g* for 30 min. The supernatant was then subjected to centrifugation at 10,000 × *g* for 30 min using a fixed-angle rotor (Fiberlite F37L-8 × 100) and compatible polycarbonate bottles. Following this step, the supernatant was sequentially filtered through a 0.45 µm and 0.22 µm membrane and ultracentrifuged at 100,000 × *g* for 2 h. The pellet was suspended in ice-cold PBS (filtered through a 0.1 µm filter), then centrifuged again under the same conditions to remove residual contaminants. Finally, the purified pellet containing sEVs was suspended in 1 mL of filtered PBS and stored at − 80 °C until further analysis.

### Preparation of human skim milk samples

To remove milk fat globules (MFG) and cellular debris, HM samples were centrifuged at 2310 × *g* for 10 min at 4 °C (Eppendorf Centrifuge 5804R, Hamburg, Germany). After centrifugation, the skim milk supernatant was collected and aliquoted (100 µL) into chromatographic vials with glass inserts. The skim milk samples were stored at –80 °C until analysis.

### Preparation of filtered human milk samples

To remove components larger than 0.22 µm from HM, the samples were passed through a 0.22 µm nylon syringe filter. The filtrates were aliquoted (100 µL) into chromatographic vials with glass inserts and stored at –80 °C until analysis.

### HIC analysis and fraction collection

HIC was performed on an Agilent Infinity II 1260 LC system equipped with a UV detector and fraction collector. MAbPac HIC-Butyl column (4.6 × 100 mm, 5 µm, non-porous butyl phase; Thermo Fisher Scientific) was applied. The column was maintained at 30 °C, the flow rate was 0.4 mL/min, and UV detection was performed at 210 nm. For fraction collection analyses, the injection volume was set to 20 µL. An inverse gradient was applied using 1.5 M ammonium sulfate with 50 mM sodium phosphate buffer pH 7.0 as mobile phase A, and 50 mM sodium phosphate buffer pH 7.0 as mobile phase B. The optimized gradient elution program was as follows: 100% solvent A from 0 to 3 min; a linear decrease from 100 to 0% A between 3 and 30 min; held at 0% A, from 30 to 50 min; ramped back to 100% A from 50 to 51 min; and maintained at 100% A from 51 to 61 min. Mobile phases were filtered through 0.45 µm nylon membrane filters prior to use. Fractions were collected using predefined retention-time windows. Retention-time windows are listed in Supplementary Table S4.

### Lipid profiling

Fractions from 10 injections were pooled to obtain sufficient material for lipidomics; lipid extraction was performed once per pooled fraction. The collected fractions were concentrated to a final volume of approximately 100 µL using Amicon Ultra-4 centrifugal filters (4 mL, regenerated cellulose membrane, 10 kDa MWCO; Millipore, Merck, Burlington, MA, USA). Lipid extraction was performed using a modified Bligh-Dyer extraction method^43,44^ with minor modifications. Briefly, fractions after concentration were transferred to borosilicate glass tubes with a PTFE cap and adjusted with deionized water to a final volume of 225 μL. Class-specific internal standards (EquiSPLASH™ LIPIDOMIX™ mixture, Avanti Polar Lipids) were added to each sample prior to lipid extraction to monitor and correct for extraction efficiency and instrumental variability (Supplementary Information, Table S5). Specifically, 10 μL of EquiSPLASH™ LIPIDOMIX™ mixture (1 µg mL⁻^1^) was added to every sample. Subsequently, 950 μL of 33:67 (*v/v*) CHCl_3_/MeOH mixture was added and vortexed for 10 s. Thereafter, 310 μL of chloroform and 310 μL of water were added, vortexed for 30 s, and centrifuged for 10 min at 3234 × g to induce phase separation. The organic phase (400 μL) was carefully collected and evaporated under a nitrogen stream at 37 °C. An extraction blank consisting of water instead of real sample was processed and analysed alongside all samples following the same extraction and LC–MS workflow to assess background signals and potential contamination. The resulting lipid extracts were reconstituted in 50 µL of 80:20 (*v/v*) MeOH/H_2_O and analyzed by reversed-phase liquid chromatography (RPLC) using 1290 LC system coupled to 6450 quadrupole-time-of-flight mass spectrometry (LC-Q-TOF–MS) with a Jet Stream Technology Ion Source (Agilent Technologies, Santa Clara, CA, USA) applying a 2.1×100 mm Kinetex EVO column (Phenomenex, Torrance, CA, USA) packed with 1.7 µm C_18_ particles fitted with 0.2 µm in-line filter. The column temperature was maintained at 60 °C, the injection volume was set at 10 µL, and the flow rate was 0.3 mL/min. Mobile phase A consisted of 5 mM ammonium formate in 80:20%(*v/v*) MeOH:H_2_O and mobile phase B was composed of 5 mM ammonium formate in 2-propanol. The gradient program was as follows: 20–40% B from 0 to 20 min, 40–60% B from 20 to 40 min, and 60–80% B from 40 to 50 min. The gradient was then increased to 100% B between 50 and 52 min, followed by a 10-min equilibration. The ESI source was operated in positive ionization mode. Data were acquired in SCAN mode across an *m*/*z* range of 200–1700 at 4 GHz. The fragmentor voltage was set to 120 V and the capillary voltage to 4000 V, with additional parameters: nebulizer gas pressure of 35 psi, drying gas flow rate of 10 L/min, and temperature of 300 °C. MS/MS analyses (*m*/*z* range between 50 and 1700) were performed under the same chromatographic and ion-source conditions, using stepped collision energies of 35 V and 80 V. The two most abundant precursor ions were fragmented and excluded from reselection for 0.3 min.

### Lipidomics data processing workflow

Lipid identification was carried out using a curated list of expected lipid species, supported by database matching (Human Metabolome Database and LipidMaps, accessed June 2025) based on an accurate m/z value (10 Δppm tolerance) and manual interpretation of the MS/MS spectra. Identification resulted in the determination of the lipid class, number of carbon atoms, and number of unsaturated bonds in fatty acid residues, as well as the presence of ether bonds instead of ester bonds in the lipid structure. Lipid species with ether-linked substituents were not differentiated regarding ether and vinyl ether bonds in position sn-1. Lipid species are reported according to the shorthand notation proposed by Liebisch et al.^45^, i.e., by class abbreviation followed by the total number of carbon atoms and double bonds in the fatty acyl chains (e.g., PC 34:1 indicates a phosphatidylcholine species with a total of 34 carbon atoms and one double bond). The suffix “O-” designates ether-linked species, and “O2” indicates the presence of two hydroxyl groups in the sphingoid base (dihydroxy sphingosine backbone). When multiple isomeric forms were detected, they are indicated as “iso 1” and “iso 2”. The diagnostic ions for the lipid class confirmation were as follows: m/z 184.0726 Da for confirmation of the SM, PC, LPC, and PC-O identity, and the characteristic neutral loss of 141.0191 Da for confirmation of the PE species. Lipid peaks were extracted and integrated with the use of Batch Targeted Feature Extraction algorithm implemented in MassHunter Mass Profinder, version B.10.00 (Agilent Technologies, Santa Clara, CA, USA). The analysis parameters were as follows: mass tolerance ± 15 ppm; retention time tolerance ± 0.3 min; ion species, + H; isotope model, common organic molecules. Data was manually verified for each lipid by assessing peak shape and ensuring that the signal-to-noise (S/N) ratio exceeded 10. Lipid quantification was based on the most abundant adduct for each lipid class, with protonated species ([M + H] +) used for the lipid classes investigated. Resulting list containing peak areas of identified lipids was further used for the relative quantification in Microsoft Excel (Microsoft, Redmond, WA, USA). Class-specific internal standards from the EquiSPLASH™ LIPIDOMIX™ mixture (Avanti Polar Lipids) were used for relative quantification: 15:0–18:1(d7) PC for PC, and PC-O species; 18:1(d7) LPC for LPC species; 15:0–18:1(d7) PE for PE species; d18:1–18:1(d9) SM for SM species; C15 Ceramide-d7 for Cer species. Lipid concentrations were calculated as:

2 $${C}_{lipid}=\frac{{A}_{lipid} \times {C}_{IS}}{{A}_{IS}} [\mu g/mL]$$

Molar concentrations were obtained by dividing by molecular mass:

3 $${C}_{Mlipid}=\frac{C{lipid} \times 1000}{M}[\mu M]$$

### Nanoparticle tracking analysis (NTA)

NTA was performed using NanoSight NS300 (Malvern Panalytical) equipped with a sCMOS camera and 488 nm laser. Camera level was set at 14, and the threshold at 5. The data was analysed with NanoSight NTA 3.4 software. Samples were thawed and diluted depending on the sample from 20 to 25,000-fold for human milk and serum-derived EVs in PBS due to the different concentration of particles. The dilution was performed with PBS filtered through a 0.1 µm size filter. For analysis, 5 individual videos of 60 s each were recorded. The background of the same PBS used for the dilution of the sample was recorded in 3 individual videos of 60 s.

### Transmission electron microscopy (TEM)

EV samples were visualized by negative-stain transmission electron microscopy. Five to 10 µL of sample were applied on the surface of formvar/carbon film coated mesh nickel grids (Electron Microscopy Sciences) and incubated for 2 min. Filter paper was used to remove liquid in excess, and 10 μL of 1% uranyl acetate was added on to the grids and left standing for 10 s, after which liquid in excess was again removed with filter paper. Visualization was carried out on a Jeol JEM-1400 transmission electron microscope at 80 kV.

### Western blot analysis

Western blotting was used as a qualitative characterization approach. The blots shown correspond to a single experiment for each sample set. Because particle numbers differed between fractions, comparable loading across all lanes was not achieved. Depending on the sample, the amount of EVs used for analysis ranged from approximately 5 × 10^6^ to 8 × 10^7^ particles. Samples were mixed with 4 × Bolt™ LDS Sample Buffer (Thermo Fisher Scientific, Waltham, MA) diluted 1:3 and heated for 10 min at 80 °C. Proteins were separated on Bolt™ 4–12% Bis–Tris Plus gels (Thermo Fisher Scientific, Waltham, MA). Electrophoresis was performed for 50 min, using a PowerEase™ 150 V Power Supply (Thermo Fisher Scientific, Waltham, MA, USA). Proteins were transferred onto nitrocellulose membranes (iBlot™ 2 Transfer Stacks, nitrocellulose, regular size, Thermo Fisher Scientific, Waltham, MA, USA) using iBlot transfer system (iBlot 2 Dry Blotting System, Thermo Fisher Scientific, Waltham, MA, USA) and the membranes were blocked in 5% (w/v) fat-free milk in 1 × TBS with 0.05% (v/v) Tween 20 (TBS-T) for one hour. Primary antibody incubations (diluted 1:250–1:1000) with 2.5% (w/v) of BSA in TBS-T buffer were carried out at 4 °C on a shaker overnight, and secondary antibody IRDye® 800CW (LI-COR Biosciences, Lincoln, NE, USA) in 1% (w/v) of BSA in TBS-T buffer (dilution 1:25,000) for 45 min at RT. The membranes were scanned and analysed using Odyssey Clx Imaging System (LI-COR Biosciences, Lincoln, NE, USA). Band intensities were quantified by densitometry using ImageJ 1.54d following background subtraction. To account for differences in EV input, all values were normalized to 1 × 10^7^ particles per sample. Data are presented as normalized band intensity (arbitrary units, a.u.). Charts with normalized data are provided in the Supplementary Information (Figure S3). Details of primary antibodies targeting EV-associated markers (CD63, flotillin-1), negative cellular markers (Calnexin), and lipoprotein-associated proteins (ApoA1) are provided in the Supplementary Information (Table S5). Protein concentration was estimated by UV absorbance at 280 nm using a NanoDrop 2000 (Thermo Fisher Scientific). The values are reported in Supplementary Figures S2, S4, S5 (panel B).

### Calculation of the vesicle hydrophobicity index (H_EV_)

An operational interfacial hydrophobicity index (*H*_EV_) was calculated for each chromatographic fraction using quantitative lipidomics data. Calculations were performed in Microsoft Excel using LC–MS-derived molar concentrations (*C*_mol,i_, µmol·L⁻^1^) for ~ 60 lipid species per fraction. The overall *HS*_EV_ for each fraction was then obtained as a weighted mean of class hydrophobicities:

4 $${H}_{\mathrm{E}\mathrm{V}}=\frac{{\sum}_{\mathrm{i}=1}^{n}\left(HG{I}_{\mathrm{c}\mathrm{l}\mathrm{a}\mathrm{s}\mathrm{s}}\times {f}_{\mathrm{c}\mathrm{l}\mathrm{a}\mathrm{s}\mathrm{s}}\times {C}_{\mathrm{m}\mathrm{o}\mathrm{l},\mathrm{i}}\right)}{{\sum}_{\mathrm{i}=1}^{n}\left({f}_{\mathrm{c}\mathrm{l}\mathrm{a}\mathrm{s}\mathrm{s}}\times {C}_{\mathrm{m}\mathrm{o}\mathrm{l},\mathrm{i}}\right)}$$

The logarithmic retention factor (log *k*_HIC_) was calculated as log((*t*_R_ − *t*_0_)/*t*_0_), where *t*_R_ denotes the fraction midpoint retention time and t_0_ was defined as the column dead time determined from the initial non-retained signal under identical gradient conditions. All calculations were performed in Microsoft Excel.

## Supplementary Information

Supplementary Information.

## Data Availability

The lipidomics raw data have been deposited in the Bridge of Knowledge repository and is available under the link https://mostwiedzy.pl/pl/open-research-data/lipidomics-raw-dataset-of-extracellular-vesicles-samples-and-fractions-obtained-by-hic-analysis,129010228128905-0?_share=4cf22584d55d19c1. Summary tables are provided in the Supplementary Information.
