# Supplementary material for: Hydrophobic interaction chromatography resolves extracellular vesicle fractions with distinct lipidomic signatures
Source: Sci Rep. 2026 May 20;16:22898. doi: 10.1038/s41598-026-50782-x (PMC13389087; doi:10.1038/s41598-026-50782-x)
Supplement: Supplementary file 1 — Supplementary Information. [file 41598_2026_50782_MOESM1_ESM.pdf]

Supplementary Information

Hydrophobic interaction chromatography resolves extracellular vesicle fractions with distinct lipidomic signatures

Michał Młynarczyk[a], Wiktoria Więckowska[a#], Mariusz Belka[b], Raphael Ewonde Ewonde[c$], Jagoda Mantej[d], Mikołaj Klimczuk[e], Felicja Gajdowska[d±], Jorge Matinha-Cardoso[f,g,h], Paula Tamagnini[h,i], Danuta Gutowska-Owsiak[e], Paulo Oliveira[g,i], Sebastiaan Eeltink[c], Weronika Hewelt-Belka[a,*]

[a] Department of Analytical Chemistry, Faculty of Chemistry, Gdańsk University of Technology, Gdańsk, Poland

[b] Department of Pharmaceutical Chemistry, Medical University of Gdańsk, Gdańsk, Poland

[c] Department of Chemical Engineering, Vrije Universiteit Brussel, Brussels, Belgium

[d] Laboratory of Experimental and Translational Allergology and Pneumology, Medical University of Gdańsk, Gdańsk, Poland

[e] Laboratory of Experimental and Translational Immunology, Intercollegiate Faculty of Biotechnology of University of Gdańsk and Medical University of Gdańsk, University of Gdańsk, Gdańsk, Poland

[f] MCbiology Doctoral Program, ICBAS – School of Medicine and Biomedical Sciences Abel Salazar, University of Porto, Porto, Portugal

[g] CIIMAR – Interdisciplinary Centre of Marine and Environmental Research, University of Porto, Matosinhos, Portugal

[h] i3S - Instituto de Investigação e Inovação em Saúde, University of Porto, Porto, Portugal

[i] Department of Biology, Faculty of Sciences, University of Porto, Porto, Portugal

*** Corresponding Author**

weronika.hewelt-belka@pg.edu.pl

Present address:

RezonBio, Gdańsk, Poland

[$] Department of Chemistry, Biosystems Research Complex, Clemson University, Clemson, South Carolina, USA

± Institute for Research in Biomedicine (IRB Barcelona), The Barcelona Institute of Science and Technology, Barcelona, Spain

Supplementary Figures and Tables

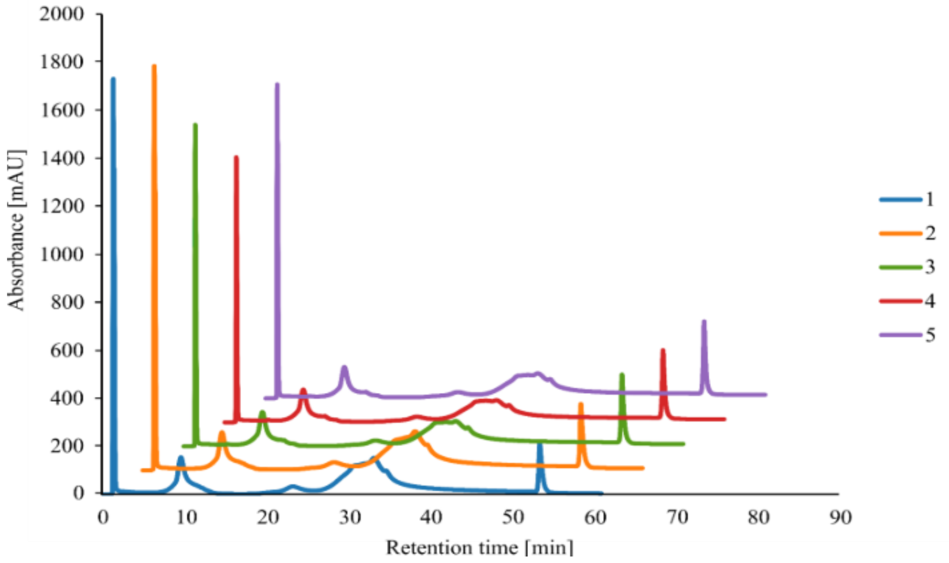

Figure S1. HIC chromatograms from repeated injections (n = 5) of a small EV (sEV) sample isolated from human milk by differential ultracentrifugation. Chromatograms are displayed as offset traces, with systematic shifts applied along both retention time (x-axis) and signal intensity (y-axis) to enhance clarity and enable visual comparison.

Table S1. Reproducibility of HIC fractionation across repeated injections (n = 5), expressed as %RSD of retention time (t_R) and UV peak area for three EV-containing fractions.

	**Fraction 1, t_R =31.9**	**Fraction 2, t_R =34.7**	**Fraction 3, t_R =53.5**
**%RSD of retention time**	3.6%	0.02%	0.04%
**%RSD of peak area**	10.8%	11.4%	8.02%

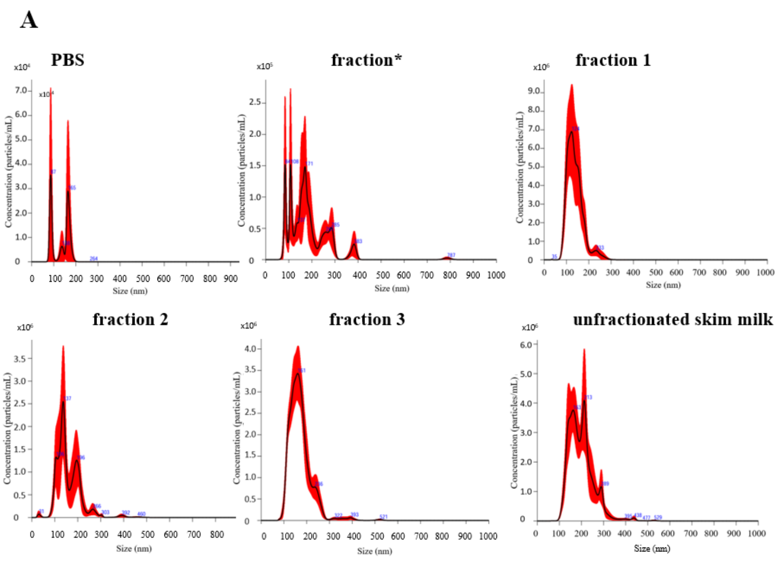

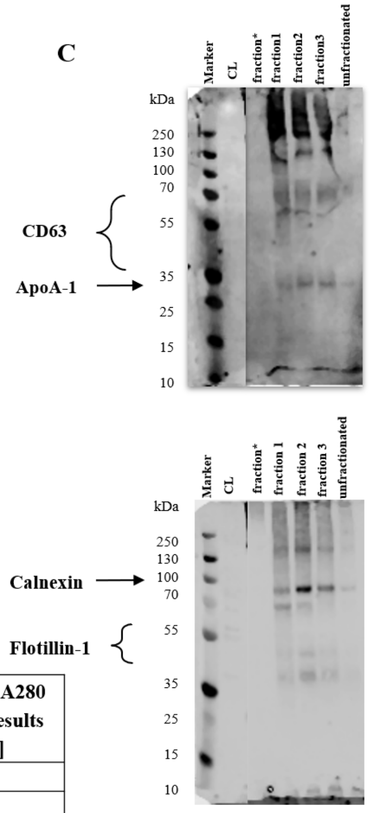

B

sample	number of sEV/mL	number of sEV/well	protein level A280 NanoDrop results [mg/mL]
**fraction***	3.02×10^8	5.01×10^6	0.003
**fraction 1**	1.04×10^{10}	8.00×10^7	1.52
**fraction 2**	3.40×10^9	5.64×10^7	0.62
**fraction 3**	6.34×10^9	8.00×10^7	0.37
**unfractionated milk-derived EV sample (after UC)**	4.00×10^{11}	8.00×10^7	18.2

Figure S2. Characterization of extracellular vesicles (EVs) isolated from human milk by differential ultracentrifugation and subsequently separated by hydrophobic interaction chromatography (HIC). Fractions 1–3 correspond to HIC-collected vesicle subpopulations, whereas the unfractionated sample represents EVs isolated from human milk by ultracentrifugation prior to HIC separation. (A) Size distribution of milk-derived EVs analyzed by nanoparticle tracking analysis (NTA). The background (PBS) was recorded using the same buffer as used for sample dilution. (B) Table summarizing the number of EVs loaded per electrophoresis lane and the total protein concentration of each sample. (C) Identification of EV-associated markers and lipoprotein-associated proteins in milk-derived EV samples by Western blot: M – protein marker; CL – cell lysate from HepG2 cells; Fractions 1–3 – milk-derived small EVs separated by HIC; Unfractionated sample – milk-derived small EVs not subjected to HIC. The fraction marked with an asterisk (fraction*) did not contain EVs detectable by transmission electron microscopy (TEM). The minimum number of vesicles per lane was ≈ 5×10^6 and the maximum ≈ 8×10^7, reflecting differences in vesicle concentration among collected fractions.

A

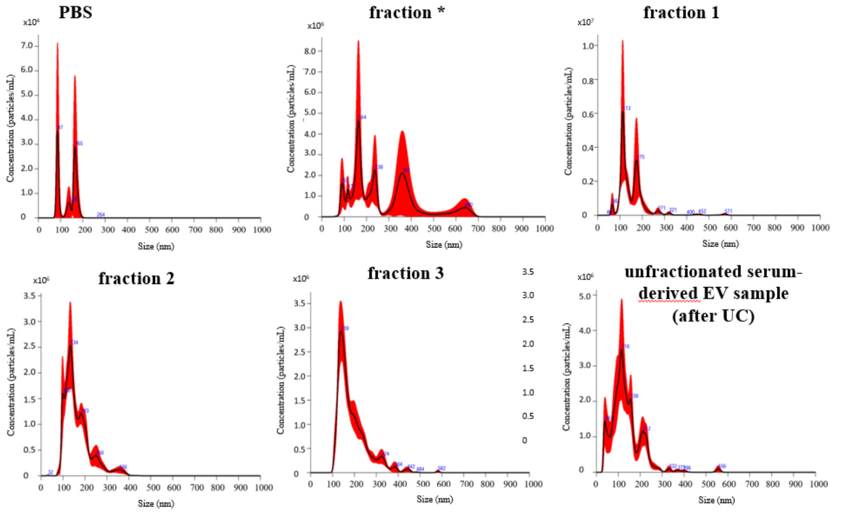

C

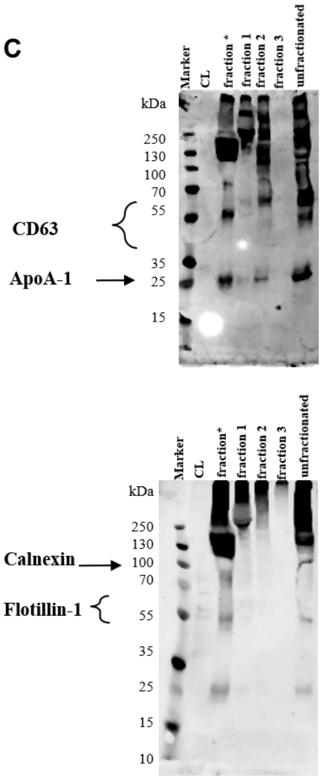

B

sample	number of sEVs/mL	number of sEVs/well	protein level A280 NanoDrop results [mg/mL]
fraction *	2.48×10^{12}	8.00×10^{7}	16.78
fraction 1	5.92×10^{9}	8.00×10^{7}	5.31
fraction 2	4.38×10^{9}	5.08×10^{7}	0.82
fraction 3	5.14×10^{9}	8.00×10^{7}	0.15
unfractionated serum-derived EV sample (after UC)	7.00×10^{9}	7.00×10^{7}	19.6

Figure S3. Characterization of extracellular vesicles (EVs) isolated from human serum by differential ultracentrifugation and subsequently separated by hydrophobic interaction chromatography (HIC). Fractions 1–3 correspond to HIC-collected vesicle subpopulations, whereas the unfractionated sample represents EVs isolated from serum by ultracentrifugation prior to HIC separation. (A) Size distribution of serum-derived EVs analyzed by nanoparticle tracking analysis (NTA). The background (PBS) was recorded using the same buffer as used for sample dilution. (B) Table summarizing the number of EVs loaded per electrophoresis lane and the total protein concentration of each sample. (C) Identification of EV-associated markers and lipoprotein-associated proteins in serum-derived EV samples by Western blot: M – protein marker; CL – cell lysate from HepG2 cells; Fractions 1–3 – serum-derived EV subpopulations separated by HIC; Original sample – serum-derived EVs not subjected to HIC. The fraction marked with an asterisk (fraction *) did not contain EVs detectable by transmission electron microscopy (TEM). The minimum number of vesicles per lane was approximately 5×10^{7} and the maximum 8×10^{7}, reflecting differences in EV concentration among collected fractions.

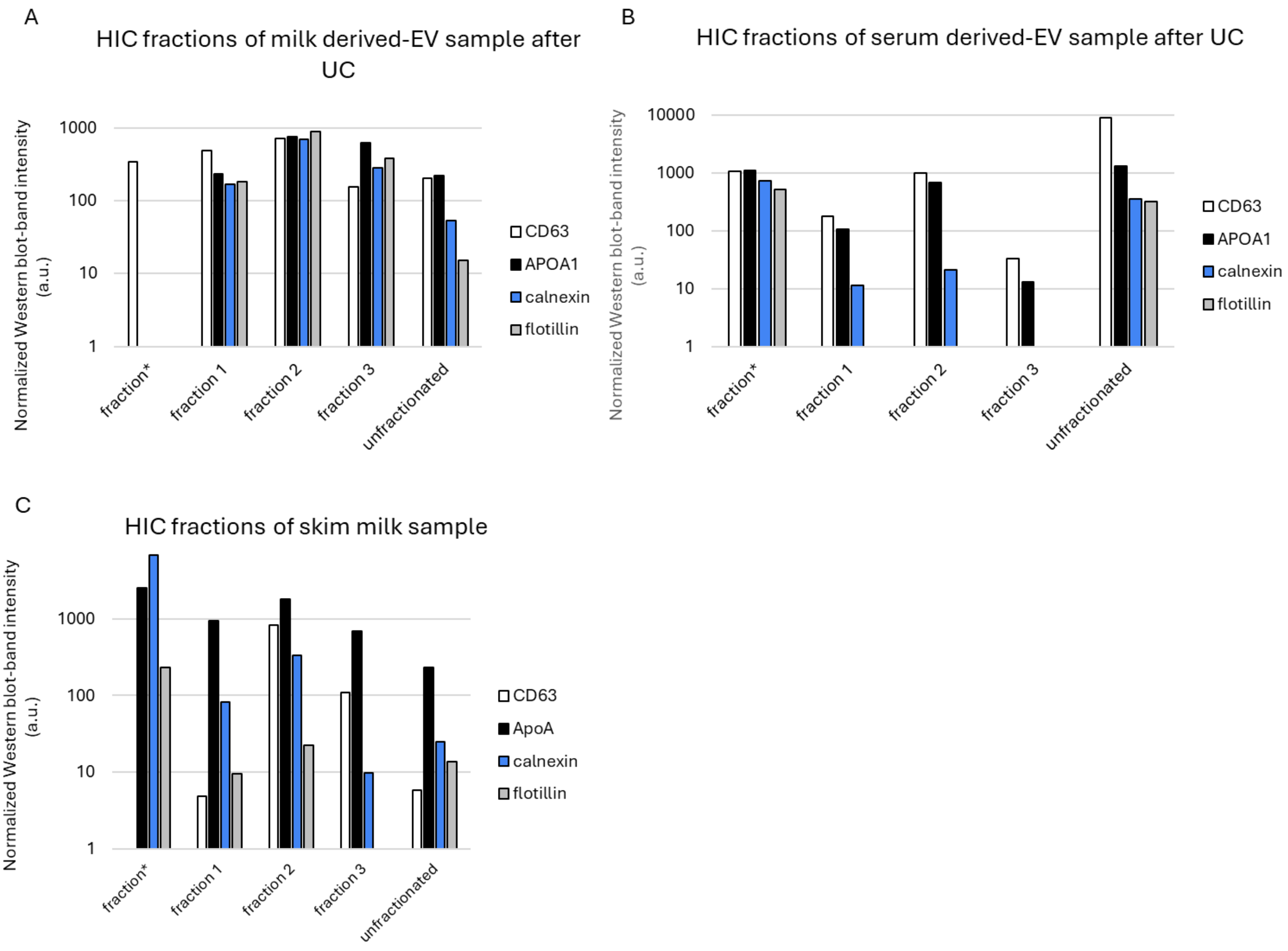

Figure S4. Densitometric analysis of CD63-, ApoA1-, Calnexin-, and Flotillin-protein band intensity detected by Western blot in fractions collected across all groups of biofluids. Band intensities were quantified using ImageJ 1.54d and normalized to 1×10^7 particles. Data are presented as arbitrary units (a.u.) on logarithmic scale. Results represent a single experiment (n = 1).

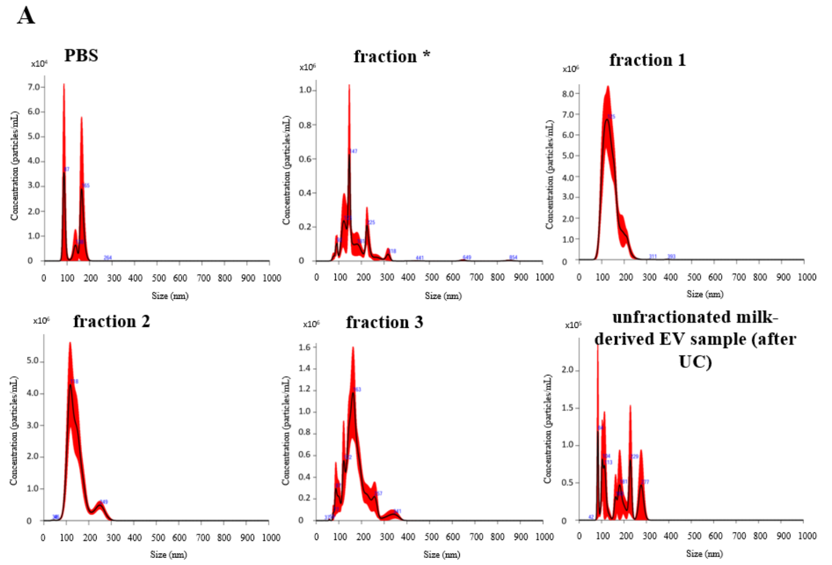

B

sample	number of sEVs/mL	number of sEVs/well	protein level A280 NanoDrop results [mg/mL]
fraction *	4.00 x 10^8	6.64 x 10^6	0.57
fraction 1	1.00 x 10^{10}	8.00 x 10^7	3.1
fraction 2	5.80 x 10^9	8.00 x 10^7	1.88
fraction 3	1.85 x 10^9	3.07 x 10^7	0.02
unfractionated skim milk	1.72 x 10^{11}	8.00 x 10^7	32.21

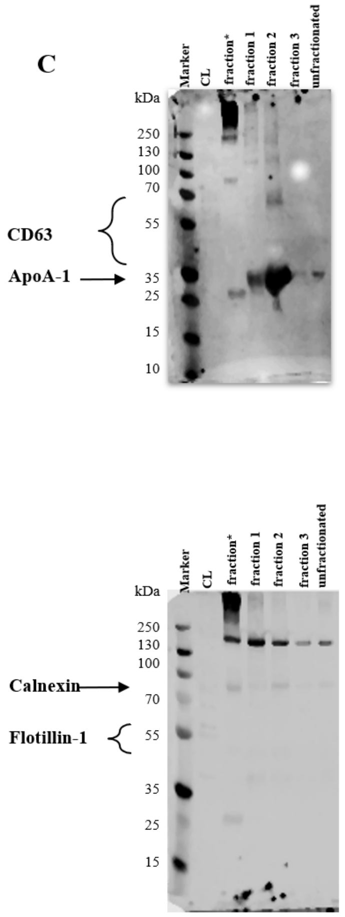

Figure S5. Characterization of extracellular vesicles (EVs) derived from human skim milk and separated by hydrophobic interaction chromatography (HIC). Skim milk was directly loaded onto the HIC column without prior EV isolation. (A) Size distribution of skim-milk–derived EV fractions and the unfractionated skim milk sample analyzed by nanoparticle tracking analysis (NTA). The background (PBS) was recorded using the same buffer as used for sample dilution. (B) Table summarizing the number of EVs loaded per electrophoresis lane and the total protein concentration of each sample. (C) Identification of EV-associated markers and lipoprotein-associated proteins in skim-milk–derived EV samples by Western blot: M – protein marker; CL – cell lysate from HepG2 cells; Fractions 1–3 – EV subpopulations collected after HIC separation; Unfractionated sample – skim milk before HIC. The fraction marked with an asterisk (fraction *) did not contain EVs detectable by transmission electron microscopy (TEM). The minimum number of vesicles per lane was approximately 6 × 10^6 and the maximum 8 × 10^7, reflecting differences in EV concentration among collected fractions.

Table S2. List of quantified lipids by RPLC-Q-TOF-MS in positive ionization mode. Fraction * denotes the fraction collected from the region marked with an asterisk in the chromatograms (no EV-like vesicles observed by negative-stain TEM under the applied imaging conditions). Lipid concentrations are reported for pooled fractions (10 injections per fraction), following concentration and extraction as described in Methods.

Lipid name	Formula	Measured neutral mass	Retention time [min]	Adduct type	Concentration [µM]									
					Serum EV Fraction *	Serum EV Fraction 1	Serum EV Fraction 2	Serum EV Fraction 3	Unfractionated serum EVs	Skim milk Fraction *	Skim milk Fraction 1	Skim milk Fraction 2	Skim milk Fraction 3	Unfractionated skim milk
**Cer 33:1; O_2**	C33H65NO3	523.4951	9.73	**$[M+H]^+$**	ND	0.0009	0.0013	0.0007	0.0003	ND	ND	0.0015	0.0008	0.0005
**Cer 32:1; O_2**	C32H63NO3	509.4806	8.74	**$[M+H]^+$**	ND	0.0013	0.0029	0.0017	ND	ND	0.0077	0.0050	0.0038	0.0082
**Cer 34:1; O_2**	C34H67NO3	537.5126	11.11	**$[M+H]^+$**	ND	0.0072	0.0195	0.0088	0.0030	ND	0.0335	0.0243	0.0113	0.0127
**Cer 42:1; O_2**	C42H83NO3	649.6376	24.45	**$[M+H]^+$**	ND	ND	0.0034	0.0034	0.0082	ND	0.0095	0.0082	0.0083	0.0163
**Cer 42:2; O_2**	C42H81NO3	647.6218	21.52	**$[M+H]^+$**	ND	ND	0.0058	0.0025	0.0075	ND	0.0191	0.0114	0.0082	0.0374
**LPC 16:0 iso 1**	C24H50NO7P	495.3327	1.79	**$[M+H]^+$**	0.0117	0.0055	0.0027	0.0025	0.0369	ND	0.0049	0.0217	ND	0.0637
**LPC 16:0 iso 2**	C24H50NO7P	495.3307	1.88	**$[M+H]^+$**	0.0182	0.0138	0.0014	0.0023	0.5216	ND	0.0327	0.1903	ND	0.1523
**LPC 18:0**	C26H54NO7P	523.3586	2.61	**$[M+H]^+$**	0.1607	0.0853	0.0081	0.0147	0.2033	ND	0.0774	0.1010	ND	0.1155
**LPC 18:1**	C26H52 NO7P	521.3489	1.86	**$[M+H]^+$**	ND	ND	0.0011	ND	0.1172	ND	0.0056	0.1333	ND	0.0140
**LPC 18:2**	C26H50NO7P	519.3329	1.62	**$[M+H]^+$**	ND	ND	0.0019	ND	0.0172	ND	ND	0.0633	ND	0.0991
**PC30:0**	C38H76NO8P	705.5313	9.27	**$[M+H]^+$**	0.0016	0.0017	0.0020	0.0015	0.0223	ND	0.0135	0.0057	0.0092	0.1159
**PC31:0**	C39H78NO8P	719.5462	9.87	**$[M+H]^+$**	ND	ND	ND	ND	0.0034	ND	0.0063	0.0027	0.0026	0.0146

**PC32:0**	C40H80NO8P	733.563	11.24	**$[M+H]^+$**	0.0074	0.0093	0.0136	0.0124	0.1071	0.0069	0.2603	0.1067	0.0812	0.4158
**PC32:1**	C40H78NO8 P	731.5466	9.68	**$[M+H]^+$**	ND	0.0014	0.0034	0.0017	0.0907	ND	0.0128	0.0086	0.0053	0.0309
**PC34:0**	C42H84NO8P	761.5943	15.03	**$[M+H]^+$**	0.0032	0.0037	0.0047	0.0043	0.0194	ND	0.1132	0.0470	0.0372	0.2000
**PC34:1**	C42H82NO8P	759.5789	11.99	**$[M+H]^+$**	0.0119	0.0262	0.0710	0.0354	1.4130	0.0113	0.2320	0.1975	0.0806	0.4265
**PC34:2**	C42H80NO8P	757.5638	10.65	**$[M+H]^+$**	0.0065	0.0213	0.0637	0.0170	1.8796	ND	0.1116	0.0884	0.0311	0.3216
**PC36:1**	C44H86NO8P	787.6093	15.86	**$[M+H]^+$**	0.0083	0.0089	0.0196	0.0116	0.2158	ND	0.0801	0.0662	0.0455	0.1141
**PC36:2 iso 1**	C44H84NO8P	785.595	13.36	**$[M+H]^+$**	0.0550	0.0179	0.0192	0.0163	0.0961	ND	0.0254	0.0220	0.0172	0.0231
**PC36:2 iso 2**	C44H84NO8P	785.595	13.57	**$[M+H]^+$**	0.0095	0.0172	0.0417	0.0107	0.7982	0.0220	0.2870	0.1285	0.0823	0.8616
**PC36:3 iso 1**	C44H82NO8P	783.5787	11.74	**$[M+H]^+$**	0.0016	0.0026	0.0067	0.0018	0.1707	ND	0.0273	0.0133	0.0066	0.0647
**PC36:3 iso 2**	C44H82NO8P	783.5788	11.34	**$[M+H]^+$**	ND	0.0033	0.0101	0.0030	0.3114	ND	0.0090	0.0078	0.0031	0.0194
**PC36:4 iso 1**	C44H80NO8P	781.5635	10.03	**$[M+H]^+$**	0.0008	0.0007	0.0021	0.0008	0.5358	ND	0.0047	0.0029	ND	0.0071
**PC36:4 iso 2**	C44H80NO8P	781.5627	10.31	**$[M+H]^+$**	0.0009	0.0048	0.0143	0.0036	0.5304	0.0004	0.0075	0.0050	0.0018	0.0150
**PC36:5**	C44H78NO8P	779.547	9.67	**$[M+H]^+$**	ND	0.0007	0.0019	0.0006	0.1032	ND	0.0022	0.0018	ND	0.0038
**PC38:2**	C46H88NO8P	813.626	17.18	**$[M+H]^+$**	0.0017	0.0016	ND	0.0012	0.0084	ND	ND	ND	ND	ND
**PC38:4**	C46H84NO8P	809.5939	13.67	**$[M+H]^+$**	0.0009	0.0034	0.0087	0.0024	0.2470	ND	0.0199	0.0109	0.0059	0.0413
**PC38:5 iso 1**	C46H82NO8P	807.5784	11.75	**$[M+H]^+$**	ND	0.0008	0.0025	ND	0.0845	ND	0.0020	ND	ND	0.0075
**PC38:5 iso 2**	C46H82NO8P	807.5772	12.99	**$[M+H]^+$**	0.0020	0.0005	0.0014	0.0004	0.0143	0.0006	0.0055	0.0022	0.0015	0.0170
**PC 38:6**	C46H80NO8P	805.563	10.62	**$[M+H]^+$**	ND	0.0013	0.0040	0.0010	0.1635	ND	0.0012	0.0017	ND	0.0034
**PC 40:6**	C48H84NO8P	833.5968	13.12	**$[M+H]^+$**	ND	ND	0.0026	0.0010	0.0458	ND	0.0033	0.0025	0.0017	0.0104
**PC 40:7**	C46H84NO8P	809.5937	13.24	**$[M+H]^+$**	ND	0.0040	0.0105	0.0026	0.2513	ND	0.0186	0.0094	0.0042	0.0413

**PC-O 32:0**	C40H82NO7P	719.5826	13.04	**[M+H]$^+$**	ND	ND	0.0014	0.0018	0.0141	ND	0.0058	0.0038	0.0020	0.0075
**PC-O 32:1**	C40H80NO7P	717.5657	12.78	**[M+H]$^+$**	ND	ND	ND	ND	0.0059	ND	0.0035	ND	ND	0.0070
**PC-O 34:1**	C42H84NO7P	745.5974	13.76	**[M+H]$^+$**	ND	ND	ND	ND	0.0299	ND	0.0098	ND	ND	0.0121
**PE 34:1**	C39H76NO8P	717.5309	13.26	**[M+H]$^+$**	ND	ND	ND	ND	ND	ND	0.1656	0.1286	ND	0.5255
**PE 34:2**	C39H74NO8P	715.5175	11.15	**[M+H]$^+$**	0.0871	0.0578	0.0541	0.0599	ND	ND	ND	0.1929	ND	1.1923
**SM 32:0; O_2**	C37H77N2O6P	676.5517	6.94	**[M+H]$^+$**	ND	ND	ND	0.0010	0.0021	ND	0.0071	0.0032	0.0033	0.0330
**SM 32:1; O_2**	C37H75N2O6P	674.5368	6.16	**[M+H]$^+$**	0.0019	0.0029	0.0067	0.0130	0.0490	ND	0.0502	0.0237	0.0252	0.2084
**SM 33:1; O_2**	C38H77N2O6P	688.5519	7.15	**[M+H]$^+$**	ND	0.0024	0.0047	0.0086	0.0273	ND	0.0151	0.0093	0.0117	0.0137
**SM 34:0; O_2**	C39H81N2O6P	704.5838	9.23	**[M+H]$^+$**	0.0022	0.0032	0.0057	0.0106	0.0292	ND	0.0399	0.0217	0.0237	0.0872
**SM 34:1; O_2**	C39H79N2O6P	702.5687	8.25	**[M+H]$^+$**	0.0235	0.0454	0.1102	0.2031	0.7384	0.0223	0.5524	0.3066	0.3336	0.8324
**SM 34:2; O_2**	C39H77N2O6P	700.5521	7.28	**[M+H]$^+$**	ND	ND	0.0068	0.0102	0.0655	ND	0.0155	ND	ND	ND
**SM 36:0; O_2 iso 1**	C41H85N2O6P	732.6171	11.93	**[M+H]$^+$**	ND	ND	ND	ND	0.0039	0.0085	0.0447	0.0182	0.0267	0.1220
**SM 36:0; O_2 iso 2**	C41H85N2O6P	732.6159	12.32	**[M+H]$^+$**	ND	ND	ND	ND	ND	ND	0.0025	ND	ND	0.0127
**SM 36:1; O_2**	C41H83N2O6P	730.5997	10.81	**[M+H]$^+$**	0.0074	0.0123	0.0231	0.0377	0.0934	0.0109	0.3915	0.1649	0.1534	1.2975
**SM 36:2; O_2**	C41H81N2O6P	728.5832	9.07	**[M+H]$^+$**	ND	ND	0.0060	0.0083	0.0432	ND	0.0250	0.0127	0.0125	0.0383

**SM 38:0; O_2**	C43H89N2O6P	760.6467	15.89	**$[M+H]^+$**	0.0004	0.0008	0.0006	0.0009	0.0014	ND	ND	ND	ND	0.0777
**SM 38:1; O_2 iso 1**	C43H87N2O6P	758.6307	14.62	**$[M+H]^+$**	0.0036	0.0051	0.0070	0.0096	0.0319	ND	0.2087	0.0840	0.0715	0.9391
**SM 38:1; O_2 iso 2**	C43H87N2O6P	758.6307	14.42	**$[M+H]^+$**	ND	0.0044	0.0081	0.0112	0.0450	ND	0.0729	0.0379	0.0375	0.2195
**SM 40:0; O_2**	C45H93N2O6P	788.6765	18.63	**$[M+H]^+$**	ND	0.0014	0.0012	0.0017	0.0032	ND	0.0147	0.0078	0.0086	0.0801
**SM 40:1; O_2 iso 1**	C45H91N2O6P	786.6619	16.91	**$[M+H]^+$**	0.0095	0.0166	0.0214	0.0280	0.1152	0.0226	0.4740	0.2043	0.1804	1.4864
**SM 40:1; O_2 iso 2**	C45H91N2O6P	786.6618	17.91	**$[M+H]^+$**	0.0095	0.0127	0.0057	0.0065	0.0232	ND	0.0510	0.0273	0.0262	0.1365
**SM 40:2; O_2 iso 1**	C45H89N2O6P	784.6455	14.36	**$[M+H]^+$**	ND	ND	0.0065	0.0058	0.0373	ND	0.0315	0.0172	0.0140	0.0549
**SM 40:2; O_2 iso 2**	C45H89N2O6P	784.6452	14.86	**$[M+H]^+$**	ND	ND	0.0091	0.0083	0.0700	ND	0.0502	0.0286	0.0231	0.1074
**SM 41:1; O_2**	C46H93N2O6P	800.6777	19.48	**$[M+H]^+$**	0.0029	0.0043	0.0072	0.0094	0.0365	ND	0.0842	0.0429	0.0417	0.2505
**SM 41:2; O_2 iso 1**	C46H91N2O6P	798.6606	15.79	**$[M+H]^+$**	ND	ND	0.0030	0.0027	0.0189	ND	0.0200	0.0115	0.0096	0.0265
**SM 41:2; O_2 iso 2**	C46H91N2O6P	798.6603	16.48	**$[M+H]^+$**	ND	ND	0.0038	0.0037	0.0190	ND	0.0198	0.0119	0.0122	0.0238
**SM 42:2; O_2**	C47H93N2O6P	812.6778	17.69	**$[M+H]^+$**	0.0053	0.0150	0.0386	0.0296	0.2355	ND	0.5480	0.2530	0.1740	0.1137
**SM 42:3; O_2**	C47H91N2O6P	810.662	15.23	**$[M+H]^+$**	ND	0.0051	0.0134	0.0076	0.1193	ND	0.0678	0.0354	0.0217	0.1188
**SM 43:2; O_2 iso 1**	C48H96N2O6P	827.6851	19.41	**$[M+H]^+$**	ND	ND	ND	ND	0.0026	ND	0.0138	0.0068	0.0063	0.0134

**SM 43:2; O_2 iso 2**	C48H96N2O6P	827.6863	19.72	**$[M+H]^+$**	0.0003	0.0003	0.0004	0.0001	0.0005	ND	0.0120	0.0049	0.0064	0.0113

ND, not detected; Cer, ceramide; LPC, lysophosphatidylcholine; PC, phosphatidylcholine; PC-O , ether-linked phosphatidylcholine; SM, sphingomyelin.

Table S3. Sample volume and corresponding particles numer of EVs subjected to lipidomics extraction.

	Human skim milk		Human serum	
	sample volume used for lipidomics [uL]	number of particles	sample volume used for lipidomics [uL]	number of particles
Fraction *	70	2.11×10^7	70	1.74×10^{11}
Fraction 1	65	6.76×10^8	85	5.03×10^8
Fraction 2	80	2.72×10^8	80	1.75×10^8
Fraction 3	110	6.97×10^8	140	7.20×10^8
Unfractionated sample	20	8.00×10^9	20	1.40×10^8

Compound Name	Serum EV Fraction 1	Serum EV Fraction 2	Serum EV Fraction 3	Unfractionated (serum EVs)	Skim milk EV Fraction 1	Skim milk EV Fraction 2	Skim milk EV Fraction 3	Unfractionated (skim milk)
**Cer 33:1; O2**	0.198%	0.182%	0.101%	0.002%	ND	0.052%	0.049%	0.004%
**Cer 32:1; O2**	0.307%	0.410%	0.258%	ND	0.173%	0.169%	0.224%	0.072%
**Cer 34:1; O2**	1.662%	2.794%	1.360%	0.021%	0.756%	0.824%	0.675%	0.112%
**Cer 41:1; O2**	ND	0.482%	0.523%	0.059%	0.214%	0.280%	0.495%	0.144%
**Cer 42:2; O2**	ND	0.831%	0.388%	0.054%	0.431%	0.388%	0.489%	0.330%
**LPC 16:1 iso 1**	1.256%	0.391%	0.389%	0.264%	0.112%	0.735%	ND	0.563%
**LPC 16:1 iso 2**	3.165%	0.204%	0.362%	3.736%	0.738%	6.450%	ND	1.345%
**LPC 18:0**	19.612%	1.170%	2.279%	1.456%	1.748%	3.425%	ND	1.020%
**LPC 18:1**	ND	0.158%	ND	0.839%	0.127%	4.518%	ND	0.124%
**LPC 18:2**	ND	0.276%	ND	0.123%	ND	2.146%	ND	0.875%
**PC 30:0**	0.387%	0.291%	0.229%	0.159%	0.306%	0.193%	0.549%	1.023%
**PC 31:0**	ND	ND	ND	0.024%	0.143%	0.090%	0.158%	0.129%
**PC 32:0**	2.146%	1.959%	1.916%	0.767%	5.876%	3.619%	4.850%	3.672%
**PC 32:1**	0.323%	0.488%	0.271%	0.650%	0.289%	0.292%	0.316%	0.273%
**PC 34:0**	0.849%	0.678%	0.667%	0.139%	2.554%	1.593%	2.222%	1.767%
**PC 34:1**	6.022%	10.204%	5.480%	10.120%	5.236%	6.694%	4.812%	3.766%
**PC 34:2**	4.891%	9.144%	2.642%	13.461%	2.519%	2.997%	1.858%	2.840%
**PC 36:1**	2.051%	2.812%	1.799%	1.546%	1.807%	2.244%	2.714%	1.007%
**PC 36:2 iso 1**	4.122%	2.757%	2.527%	0.688%	0.574%	0.747%	1.027%	0.204%
**PC 36:2 iso 2**	3.946%	5.985%	1.654%	5.717%	6.476%	4.356%	4.911%	7.609%
**PC 36:3 iso 1**	0.589%	0.956%	0.274%	1.223%	0.616%	0.450%	0.395%	0.571%
**PC 36:3 iso 2**	0.770%	1.455%	0.465%	2.230%	0.203%	0.264%	0.187%	0.172%
**PC 36:4 iso 1**	0.165%	0.297%	0.120%	3.837%	0.107%	0.099%	ND	0.063%
**PC 36:4 iso 2**	1.113%	2.060%	0.561%	3.799%	0.170%	0.170%	0.109%	0.133%
**PC3 6:5**	0.157%	0.279%	0.099%	0.739%	0.050%	0.061%	ND	0.033%
**PC 38:2**	0.373%	ND	0.189%	0.060%	ND	ND	ND	ND
**PC 38:4**	0.783%	1.253%	0.377%	1.769%	0.449%	0.371%	0.353%	0.365%
**PC 38:5 iso 1**	0.194%	0.352%	ND	0.605%	0.044%	ND	ND	0.066%
**PC 38:5 iso 2**	0.105%	0.203%	0.063%	0.102%	0.124%	0.074%	0.091%	0.150%
**PC 38:6**	0.290%	0.568%	0.162%	1.171%	0.028%	0.058%	ND	0.030%
**PC 40:6**	0.000%	0.368%	0.150%	0.328%	0.075%	0.084%	0.100%	0.092%
**PC 40:7**	0.909%	1.511%	0.400%	1.800%	0.420%	0.318%	0.253%	0.365%
**PC-O 32:0**	ND	0.205%	0.278%	0.101%	0.131%	0.129%	0.120%	0.066%
**PC-O 32:1**	ND	ND	ND	0.042%	0.078%	ND	ND	0.061%
**PC-O 34:1**	ND	ND	ND	0.214%	0.222%	ND	ND	0.107%
**PE 34:1**	ND	ND	ND	ND	3.737%	4.359%	ND	4.641%
**PE 34:2**	13.303%	7.771%	9.289%	ND	ND	6.541%	ND	10.529%
**SM 32:0;O2**	ND	ND	0.148%	0.015%	0.161%	0.109%	0.198%	0.291%
**SM 32:1;O2**	ND	0.959%	2.022%	0.351%	1.134%	0.804%	1.505%	1.841%
**SM 33:1;O2**	0.542%	0.669%	1.337%	0.195%	0.340%	0.314%	0.698%	0.121%
**SM 34:0;O2**	0.734%	0.821%	1.643%	0.209%	0.900%	0.735%	1.414%	0.770%
**SM 34:1;O2**	10.445%	15.827%	31.476%	5.288%	12.467%	10.393%	19.915%	7.351%
**SM 34:2;O2**	ND	0.972%	1.582%	0.469%	0.349%	ND	ND	ND
**SM 36:0;O2 iso 1**	ND	ND	ND	0.028%	1.009%	0.616%	1.596%	1.078%
**SM 36:0;O2 iso 2**	ND	ND	ND	ND	0.057%	ND	ND	0.112%
**SM 36:1;O2**	2.834%	3.322%	5.839%	0.669%	8.835%	5.591%	9.158%	11.458%
**SM 36:2;O2**	ND	0.855%	1.290%	0.309%	0.565%	0.431%	0.749%	0.338%
**SM 38:0;O2**	0.176%	0.086%	0.133%	0.010%	ND	ND	ND	0.687%
**SM 38:1;O2 iso 1**	1.169%	1.007%	1.488%	0.228%	4.710%	2.848%	4.267%	8.293%
**SM 38:1;O2 iso 2**	1.004%	1.167%	1.738%	0.322%	1.645%	1.286%	2.237%	1.939%
**SM 40:0;O2**	0.330%	0.166%	0.257%	0.023%	0.332%	0.263%	0.513%	0.707%
**SM 40:1;O2 iso 1**	3.808%	3.071%	4.343%	0.825%	10.697%	6.926%	10.769%	13.127%
**SM 40:1;O2 iso 2**	2.931%	0.812%	1.001%	0.166%	1.150%	0.924%	1.565%	1.205%
**SM 40:2;O2 iso 1**	ND	0.927%	0.906%	0.267%	0.710%	0.583%	0.838%	0.485%
**SM 40:2;O2 iso 2**	ND	1.311%	1.294%	0.501%	1.133%	0.971%	1.381%	0.948%
**SM 41:1;O2**	0.982%	1.031%	1.461%	0.261%	1.900%	1.454%	2.492%	2.212%
**SM 41:2;O2 iso 1**	ND	0.430%	0.413%	0.135%	0.451%	0.388%	0.571%	0.234%
**SM 41:2;O2 iso 2**	ND	0.540%	0.574%	0.136%	0.447%	0.403%	0.731%	0.210%
**SM 42:2;O2**	3.460%	5.549%	4.594%	1.687%	12.368%	8.578%	10.391%	1.004%
**SM 42:3;O2**	1.162%	1.931%	1.176%	0.855%	1.529%	1.201%	1.294%	1.049%
**SM 43:2;O2 iso 1**	ND	ND	ND	0.018%	0.311%	0.230%	0.376%	0.118%
**SM 43:2;O2 iso 2**	0.071%	0.054%	0.013%	0.004%	0.271%	0.166%	0.385%	0.100%

0.002 **31.476**

Figure S6. Lipidomic heatmaps showing relative abundances (% of total lipids) of individual lipid species across HIC-separated EV-containing fractions and the corresponding unfractionated (prior to HIC separation) serum and skim milk samples. Color gradients represent relative abundance from lowest (blue) to highest (red). ND, not detected; Cer, ceramide; LPC, lysophosphatidylcholine; PC, phosphatidylcholine; PC-O, ether-linked phosphatidylcholine; SM, sphingomyelin.

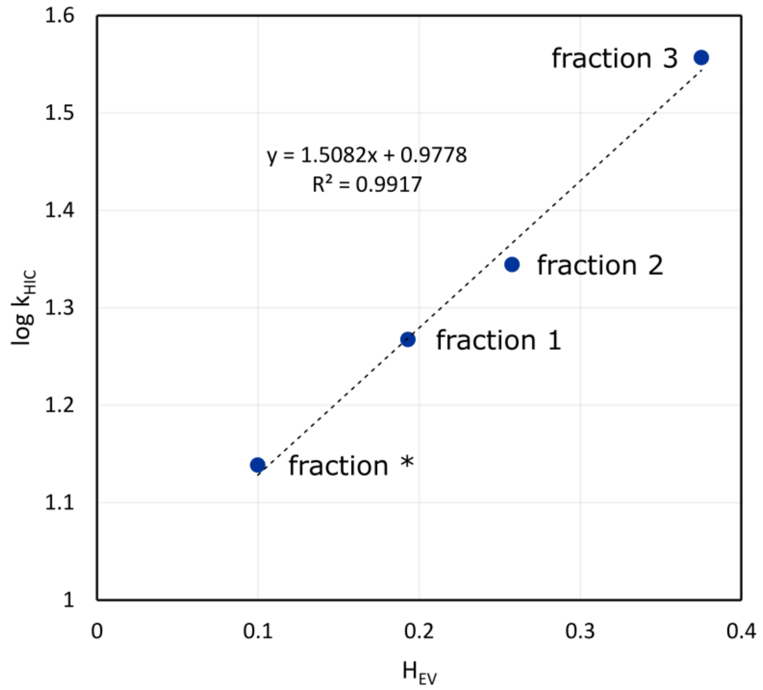

Figure S7. Correlation between the operational interfacial hydrophobicity index, (H_{EV}) and logarithm of retention factor in HIC ($log\ k_{\mathrm{HIC}}$) for four separated fractions (fraction *, fraction 1, fraction 2, fraction 3) from human serum-derived small EVs. *log k*HIC was calculated using the midpoint retention time of each fraction.

Table S4. Retention time windows used for fraction collection during hydrophobic interaction chromatography (HIC) separations.

Human milk EV sample and skim milk	Time [min]		Serum EV sample	Time [min]
Fraction *	21.80 - 24.92		Fraction *	19.06 - 23.91
Fraction 1	28.82 - 33.51		Fraction 1	27.14 - 29.36
Fraction 2	34.37 - 36.87		Fraction 2	32.19 - 35.25
Fraction 3	53.27 - 54.92		Fraction 3	53.31 - 54.56

Table S5. Percent Relative standard deviation (%RSD) of class-specific internal standards across two independent LC–MS analytical sequences. Internal standards were added prior to extraction.

Compound Name	%RSD	%RSD
	Sequence 1	Sequence 2
**C15 Ceramide-d7**	15.43%	3.01%
**18:1(d7) Lyso PC**	22.03%	39.98%
**15:0-18:1(d7) PE**	12.86%	19.82%
**d18:1-18:1(d9) SM**	16.28%	11.79%
**15:0-18:1(d7) PC**	15.08%	14.05%

Table S6. List of the antibodies used in the study.

Target antigen	Clone	Tag/fluorophore	Manufacturer	Cat. no.	Dilution
**CD63**	E12	-	Santa Cruz Biotechnology	sc-365604	1:1000
**Calnexin**	AF18	-	Santa Cruz Biotechnology	sc-23954	1:250
**Flotillin 1**	18/Flotillin-1	-	BD Biosciences	610820	1:500
**ApoA1**	B-10	-	Santa Cruz Biotechnology	sc-376818	1:250
